# High-Quality Complete and Draft Genome Sequences for Three *Escherichia* spp. and Three *Shigella* spp. Generated with Pacific Biosciences and Illumina Sequencing and Optical Mapping

**DOI:** 10.1128/genomeA.01384-17

**Published:** 2018-01-04

**Authors:** Max R. Schroeder, Phalasy Juieng, Dhwani Batra, Kristen Knipe, Lori A. Rowe, Mili Sheth, Peyton Smith, Lisley Garcia-Toledo, Vladimir N. Loparev, Rebecca L. Lindsey

**Affiliations:** a Biotechnology Core Facility Branch, Division of Scientific Resources, Centers for Disease Control and Prevention, Atlanta, Georgia, USA; b Oak Ridge Institute for Science and Education, Oak Ridge, Tennessee, USA; c Enteric Diseases Laboratory Branch, Division of Foodborne, Waterborne and Environmental Diseases, Centers for Disease Control and Prevention, Atlanta, Georgia, USA

## Abstract

*Escherichia* spp., including *E. albertii* and *E. coli*, *Shigella dysenteriae*, and *S. flexneri* are causative agents of foodborne disease. We report here reference-level whole-genome sequences of *E. albertii* (2014C-4356), *E. coli* (2011C-4315 and 2012C-4431), *S. dysenteriae* (BU53M1), and *S. flexneri* (94-3007 and 71-2783).

## GENOME ANNOUNCEMENT

Out of 317 strains submitted for whole-genome mapping, we selected 6 *Escherichia* strains that exhibited a prominent DNA degradation phenotype using the standard OpGen DNA extraction protocol. Isolate 2011C-4315 was previously PacBio sequenced (1), but the sequence released here is of higher quality due to additional Pacific Biosciences (PacBio) and Illumina sequencing using new technology. Long, high-quality DNA preparations for optical mapping were obtained after short inactivation with Stabilizor T1 (Denator, Gothenburg, Sweden).

Genomic DNA was extracted by using commercial protocols (MasterPure, Epicentre, Chicago, IL, USA; and ArchivePure, 5 Prime, Gaithersburg, MD, USA). DNA was used to generate either 10-kb or 20-kb libraries with the SMRTbell template prep kit version 1.0 (PacBio, Menlo Park, CA, USA). All 20-kb libraries were size selected with BluePippin (Sage Scientific, Beverly, MA, USA). Libraries were bound to polymerase using the DNA/polymerase binding kit P5 or P6v2 and were then loaded on single-molecule real-time (SMRT) cells and sequenced with C3 (P5 polymerase) or C4v2 chemistry (P6v2 polymerase) for 270-min (10-kb libraries) or 360-min (20-kb libraries) movies on the RSII instrument (PacBio). Sequence reads were assembled *de novo* using the Hierarchical Genome Assembly Process (HGAP3) from the SMRT Analysis Software suite (PacBio) (2). Aliquots of DNA were also used for MiSeq sequencing according to the manufacturer’s protocols (Illumina, San Diego, CA, USA). DNA samples were sheared to a mean size of 600 bp utilizing a Covaris LE220 focused ultrasonicator (Covaris Inc., Woburn, MA, USA) and cleaned with AMPure (Beckman Coulter, Inc., Indianapolis, IN, USA). Dual-indexed sequencing libraries were prepared with NEBNext ultra DNA library prep kits for Illumina (New England Biolabs, Ipswich, MA, USA), and barcoding indices were synthesized in-house. The resulting libraries were analyzed for size and concentration, pooled, and denatured for loading onto a flowcell for cluster generation. Sequencing was performed using 2 × 250-cycle paired-end sequencing with an Illumina MiSeq reagent kit version 2 on the Illumina MiSeq platform. The sequence reads were filtered for read quality, base called, and demultiplexed utilizing Bcl2fastq version 1.8.4. Illumina reads were assembled *de novo* using CLC Genomic Workbench version 9. The PacBio-generated contigs were corrected by aligning Illumina reads using Pilon version 1.21 (3). Whole-genome optical maps were created using NcoI or AflII digestion with the Argus platform (OpGen, Gaithersburg, MA, USA), and the genomic sequences were verified using corresponding *in silico* restriction enzyme maps.

The accession numbers and assembly metrics for each combined PacBio and Illumina assembly that was confirmed with optical maps are listed in Table 1. A single chromosomal contig was generated for each isolate with 200× to 500× coverage, and all sequences but one were determined to be circular with overlapping ends. Isolate 71-2783 was not circularized, as it contains an unresolved collapsed repeat region. The plasmid contigs associated with these isolates had 200× to 600× coverage, had overlapping ends, and were closed.

**TABLE 1:** Accession numbers and assembly metrics of six enteric complete and draft whole-genome sequences

Species	Isolate no. (reference)	Serotype	Chromosomal GenBank accession no.	Genome size (bp)	G+C content (%)	Associated plasmid size (bp) (GenBank accession no.)
*E. albertii*	2014C-4356 (EA-3) (4)	None	CP024282	4,852,165	49.80	40,461 (CP024283)
						59,626 (CP024284)
						127,606 (CP024285)
						113,727 (CP024286)
						124,142 (CP024287)
						19,118 (CP024288)
*E. coli*	2011C-4315 (1)	O153:H2	CP024479	5,336,099	50.75	77,062 (CP024480)
						105,489 (CP024481)
*E. coli*	2012C-4431	O178:H19	CP024289	5,074,559	50.89	36,473 (CP024290)
						111,697 (CP024291)
						85,054 (CP024292)
*S. dysenteriae*	BU53M1	1	CP024466	4,409,083	51.26	54,993 (CP024467)
						115,922 (CP024468)
						184,894 (CP024469)
*S. flexneri*	94-3007	7b	CP024473	4,533,699	50.94	69,554 (CP024474)
						82,833 (CP024475)
						220,282 (CP024476)
*S. flexneri^a^*	71-2783	3a	CP024470	4,834,497	50.93	97,011 (CP024470)
						159,299 (CP024471)

a Denotes draft sequence due to an unresolved collapsed repeat region.

Future publications will report on additional analyses of these complete and draft genomes.

### Accession number(s).

The whole-genome shotgun projects reported here have been deposited in DDBJ/ENA/GenBank under the accession numbers listed in Table 1. The versions described in this paper are the first versions, except for 2011C-4315, which is the second version.
